# Understanding medical cannabis use internationally: Why definitions and context matter

**DOI:** 10.1111/add.70117

**Published:** 2025-07-01

**Authors:** Myfanwy Graham, Rosalie Liccardo Pacula, Seema Choksy Pessar, Yimin Ge, Alexandra F. Kritikos, Wayne Hall, David Hammond

**Affiliations:** ^1^ Monash Addiction Research Centre Monash University Melbourne VIC Australia; ^2^ Centre for Drug Repurposing and Medicines Research Hunter Medical Research Institute Newcastle NSW Australia; ^3^ Schaeffer Center for Health Policy & Economics University of Southern California Los Angeles CA USA; ^4^ Johns Hopkins University Bloomberg School of Public Health Baltimore MD USA; ^5^ NORC University of Chicago Cambridge MA USA; ^6^ Centre for Youth Substance Abuse Research University of Queensland Brisbane QLD Australia; ^7^ School of Public Health Sciences University of Waterloo Waterloo ON Canada

**Keywords:** cannabis policy, medical cannabis, methodology, public health, recreational cannabis, surveys and questionnaires

## Abstract

**Aims:**

To identify variation in identification of medical consumers using alternative self‐reported measures and assess whether differences in these rates exist across jurisdictions with different medical policy approaches using evidence from an international study on cannabis use.

**Design:**

Secondary analysis of wave 4 (2021) of the International Cannabis Policy Study (ICPS) cross‐sectional survey.

**Setting:**

United States, Canada and Australia.

**Participants:**

16 951 (USA 10 472; CAN 5935; AUS 544) respondents who completed the survey and reported past year cannabis use across the three jurisdictions.

**Measurements:**

Four different medical cannabis use measures were available, and rates of each were estimated using logistic regression methods that adjusted for age, gender, education and ethnicity. Medical cannabis use measures included potentially authorized use (i.e. involving a licensed health professional recommendation, authorization or prescription), pharmaceutical use (i.e. involving a pharmaceutical‐grade product), therapeutic use (i.e. to manage physical or mental health conditions) and self‐identified medical cannabis use. Country‐specific differences were compared and discussed in light of measure and differing cannabis policies.

**Findings:**

In wave 4 of the ICPS, 34.0% reported any past year cannabis use, but rates of medical use differed significantly according to the specific question. Far more individuals reported therapeutic use in the past year across all countries [77.3%; 95% confidence interval (CI) = 76.4%–78.2%] than any other measure of medical use. While just over one quarter (28.2%; 95% CI = 27.3%–29.2%) self‐identified as a medical user, fewer reported being potentially authorized (22.8%; 95% CI = 22.0%–23.7%) or having a pharmaceutical prescription from a medical professional (12.3%; 95% CI = 11.6%–13.0%). Australians (27.2%; 95% CI = 23.0%–31.4%) and Americans (25.9%; 95% CI = 24.6%–27.2%) were more likely to report potentially authorized use than Canadians (17.3%; 95% CI = 16.1%–18.4%), but only Australians (27.4%; 95% CI = 23.6%–31.2%) reported high levels of prior use of a pharmaceutical‐grade cannabinoid.

**Conclusions:**

In the International Cannabis Policy Study, the proportion of respondents (adjusted for demographic factors) who reported medical use varied depending on the measures used within and between countries.

## RATIONALE

Global interest in the medical use of cannabis has grown significantly in recent years despite limited supportive evidence from randomized control trials (RCTs). Studies of medical use using real‐world data in naturalistic settings have struggled to address differences in the products used [1, 2], variability in the cannabinoids present in similarly named products [3, 4], differences in clinical treatment guidance [5] and differences among jurisdictions in which medical conditions qualify for cannabis use [1, 6, 7]. Surveys of the prevalence of medical cannabis use are affected by each of these factors and an additional one: whether the patient considers their use to be medical.

This study uses data from the International Cannabis Policy Study (ICPS) [8] to investigate how the specific questions posed about medical use can influence response rates in nationally representative samples of adults from the USA, Canada and Australia, three countries with distinctly different medical markets.

In the USA, 76% of states have legalized medical cannabis use as of June 2024, and more than half of those have also legalized non‐medical ‘adult use’ [9]. USA federal law, however, continues to prohibit the use of cannabis for either purpose. In Canada, legal access to medical cannabis was established in 1999, although the number of individuals seeking medical authorizations has decreased substantially since the federal legalization of non‐medical cannabis use in 2018 [10]. Medicinal cannabis use was legalized federally in Australia in 2016, although non‐medical cannabis use remains illegal and states differ in the penalties imposed for non‐medical use [11].

This study assessed how survey participants responded to different questions related to medical cannabis use within each country as well as across countries. To take account of national differences in terminology (e.g. medical marijuana, medicinal cannabis, medical cannabis products, etc.), the term ‘medical cannabis’ will be used throughout this article to refer to the medical use of a product that contains cannabinoids. The term ‘marijuana’ will be used only in reference to specific questions in the ICPS survey that used this term.

## DATA, MEASURES & METHODS

### Study design and data

Cross‐sectional data from Wave 4 of the 2021 ICPS were collected via self‐completed web‐based surveys administered from September through October 2021. The target population are individuals aged 16–64 years residing in households. Samples were obtained via the Nielsen Consumer Insights Global Panel and partner panels. For the ICPS survey, Nielsen draws stratified random samples from its online panels, using quotas based on age (16–64 years) and state/province of residence. These respondents receive remuneration to participate in ICPS in accordance with their panel's incentive structure upon survey completion. Post‐stratification sample weightings are constructed based on age, sex and education to generate nationally representative samples for each country. Surveys are conducted in English (in the USA, parts of Canada and Australia) or French (in parts of Canada). More than 49 950 individuals responded to the ICPS Wave 4 survey across the three countries, of which two‐thirds (64.4%) responded to questions on past‐year cannabis use.

The study received ethics clearance from the University of Waterloo Research Ethics Committee (ORE#31330). For further details on study methods, see the technical reports and methodology article [8, 12].

### Medical cannabis use measures

**FIGURE 1:**
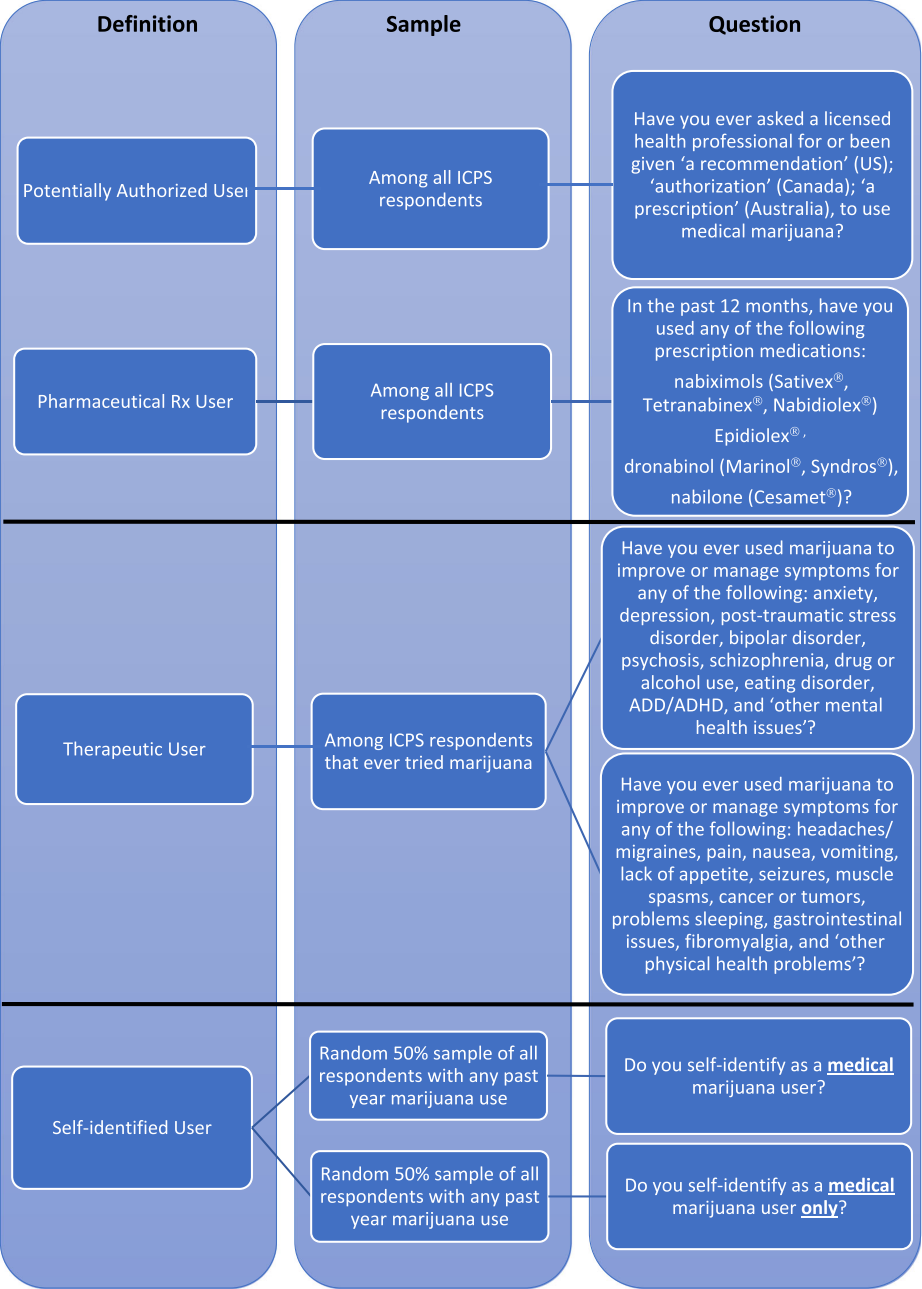
Medical cannabis measures derived from the International Cannabis Policy Study (ICPS) survey.

The survey contains multiple items pertaining to medical use, allowing us to construct four different measures of medical cannabis use (for the exact questions posed, see Figure 1). The first two, asked of all respondents, focused on medical use through a healthcare provider. *Potentially authorized* users are defined as having ever asked for or received a recommendation, authorization or prescription to use medical marijuana from a licensed health professional. *Pharmaceutical* *Rx* users are defined as those who report being prescribed a pharmaceutical‐grade cannabis medication in the past 12 months, including dronabinol, Epidiolex®, nabilone, nabiximols or a ‘prescribed cannabis‐based medicinal product’. The third measure, *therapeutic use*, was only asked of respondents who report ever trying cannabis and was intended to capture use to manage symptoms from specified mental or physical health conditions. Finally, *self‐identified medical use* was based on a question posed of past‐year cannabis users who were asked whether they self‐identified as a medical cannabis user. For this last measure, the question was randomized in the survey so that 50% of those surveyed were asked if they *only* viewed themselves as taking cannabis for medical purposes, whereas the other 50% were asked if they consider themselves a ‘medical marijuana user’. Responses of ‘do not know’ were coded as non‐medical use and ‘refused to answer’ were coded as missing for our analyses. These four medical use measures are not mutually exclusive (e.g. a *self‐identified* individual may also be an individual reporting *therapeutic use*).

### Data analysis

We first report the number of respondents who reported using cannabis in the past year, and then the share reporting medical use. Because there are systematic differences in populations represented across countries (see Table S1), the share of respondents for each of the four medical use measures are estimated using multinomial logistic regression methods to adjust for gender, age, race, education, country and sampling weights, using STATA 17.0 (StataCorp LLC, College Station, TX, USA). Age‐, gender‐, ethnicity‐ and education‐adjusted predicted response rates of all combinations of these four medical measures are generated from these models.

## RESULTS

Our analytic sample included 16 951 respondents who reported past‐year cannabis use from Australia (3.3%; *n* = 544), Canada (36.2%; *n* = 5935) and the USA (60.5%; *n* = 10 472). It omitted 16 respondents who did not provide complete information on age, gender, race/ethnicity or education (*n* = 16).

**FIGURE 2:**
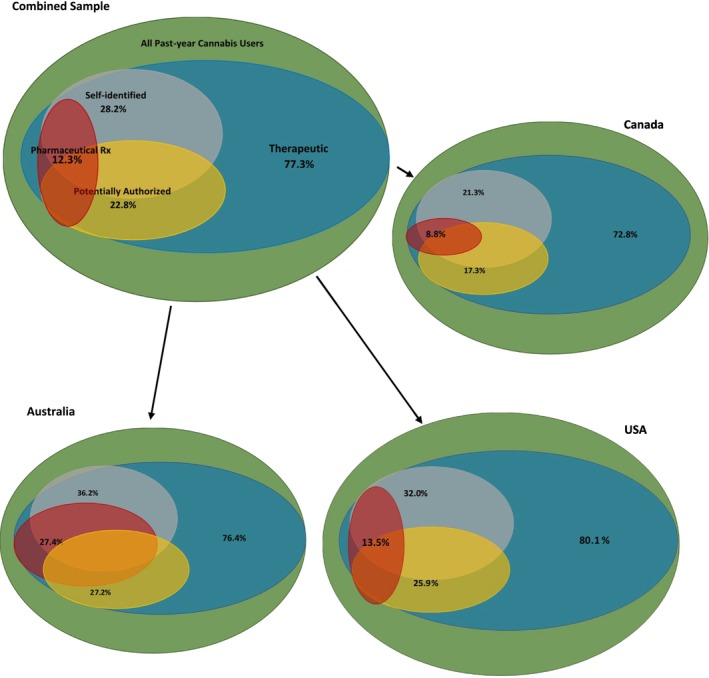
Multinomial logistic model estimates of adjusted medical use measures in the International Cannabis Policy Study (ICPS) Wave 4 (2021) data.

Figure 2 presents Venn diagrams showing the adjusted shares of past‐year cannabis users who reported medical use in the combined sample and then in each country. The values shown in Figure 2 are provided in Table S2 and the confidence intervals are provided in Table S3.

There were three overlapping categories of medical use that we were insufficiently powered to estimate among past‐year users, specifically: (i) those who were *potentially authorized* and an *R*x user, but did not *self‐identify* as a medical user or report *therapeutic use* (*n* = 3); (ii) those who reported being an *Rx* user and who *self‐identified* as a medical consumer, but who did not report being *potentially authorized* or *therapeutic use* (*n* = 3); and (iii) those who identified as *potentially authorized*, a *Rx* user and *self‐identify* as a medical user, but did not report *therapeutic use* (*n* = 15).

In the combined data from all three countries, far more past‐year cannabis users (77.3%; 95% CI = 76.4%–78.2%) reported the *therapeutic use* of cannabis, even though less than a quarter (22.8%; 95% CI = 22.0%–23.7%) reported being *potentially authorized* and less than a third *self‐identified* as a medical cannabis user (28.2%; 95% CI = 27.3%–29.2%). A much smaller percentage (12.3%; 95% CI = 11.6%–13.0%) reported being an *Rx* user. This pattern of use was similar for all countries.

The likelihood of someone *self‐identifying* as a medical cannabis user was higher in the USA (32.0%; 95% CI = 30.6%–33.4%) and Australia (36.2%; 95% CI = 31.6–40.8%) than in Canada (21.3%; 95% CI = 20.0%–22.5%). Additionally, respondents in Australia were more likely (27.4%; 95% CI = 23.6%–31.2%) than Americans or Canadians to report being a *Rx* user (13.5% and 8.8%, respectively).

In the combined sample, almost everyone who reported being *potentially authorized*, *Rx* use or *self‐identified medical use* also reported *therapeutic use*. Only very small groups of respondents (< 5%) reported one of these three categories in the absence of reporting *therapeutic use* (see Table S2). Those reporting *self‐identified medical use* had the greatest overlap with those reporting *therapeutic use* (35.5%), although in Australia there was a lot more overlap between *therapeutic use* and the other two types of medical use as well. Only 32.3% (95% CI = 28.0%–36.5%) of Australian respondents report *therapeutic use* alone. This was substantially lower than reported in the USA (38.8%; 95% CI = 37.4%–40.2%) and was somewhat lower than reported in Canada (43.6%%; 95% CI = 42.1%–45.2%).

Importantly, in all three countries fewer than half of those who reported *therapeutic use* to manage mental or physical health symptoms had engaged with a healthcare provider. We see greater engagement of those reporting *therapeutic use* with healthcare providers in Australia (34.1%–35.5%) than in the USA (11.7%–22.9%) or Canada (16.6%–31.4%).

## DISCUSSION

The findings from this study illustrate the challenges in estimating the prevalence of medical cannabis use within and between jurisdictions given different ways of identifying it. Jurisdictions vary in the conditions and products that can be used for medicinal purposes [13]. Hence, even standardized questions will be interpreted through these jurisdictional differences, as will the ways in which medical cannabis is accessed. This study shows clearly that in these three jurisdictions people who use cannabis therapeutically differed in their engagement with healthcare providers and their use of pharmaceutical‐grade products. While we consistently find that a relatively small share of people who self‐identify as medical users or acknowledge using cannabis for therapeutic purposes engage with a medical provider, it was even less common in Canada where cannabis is also available recreationally [14].

The findings from this study suggest that if researchers focus on patients who engage with a medical provider about medical cannabis use, this will capture only a small proportion of people who self‐identify as a medical cannabis user or use cannabis therapeutically in every jurisdiction. Despite the regulatory differences between countries, self‐identified medical cannabis use represented 21.3%–36.2% of past‐year use within each country. As in prior research, we found a far greater proportion of the population reported therapeutic use rather than accessing medical cannabis via regulated pathways, even when legally allowed to do so [10, 14]. Studies focused on medically supervised populations (i.e. those who use cannabis through the traditional healthcare system) will accordingly miss a large share of individuals who use cannabis for medical reasons. These variations across measures within populations occur despite the requirement in each jurisdiction for consultation with a medical provider.

The study also revealed that the extent to which the medical use of cannabis overlaps with recreational use depends on how medical use is defined, regardless of country. There was substantial overlap (>70%) between *therapeutic use* and past‐year recreational use in all three countries, with a substantially lower degree of overlap (<30%) between *potentially authorized*, *Rx* use or *self‐identified medical use* and past‐year use. Differences in federal policies toward medical use and adult use of cannabis are likely to influence the proportion of respondents who reported seeking medical authorization or who self‐identified as a medical user. These differences might also reflect underlying population differences in the willingness to engage with the healthcare system or try pharmaceutical therapies. For example, Australians were far more likely to use a pharmaceutical‐grade product (27.4%) than Americans (13.5%) or Canadians (8.8%).

This study has several limitations. First, the ICPS sample is derived from the Nielsen commercial marketing panels that represent household purchasers, not cannabis consumers specifically. Moreover, they were obtained using both probability‐ and non‐probability‐based sampling due to reliance on Nielsen consumer panels, which could limit the generalizability. For example, the ICPS has a greater proportion of female participants. However, the weighted ICPS results produced estimates of past‐year cannabis use that were generally consistent with that in nationally representative samples of households in Australia, Canada and the USA [8, 12]. Finally, this is a large international study and policy differences may have affected individual responses to specific questions; jurisdictional differences in marketing restrictions, compliance and enforcement may influence motivations for using cannabis medically.

## CONCLUSION

Differences in the measurement of medical cannabis use may arise from differences in questions used to assess medical use and differences in regulations between jurisdictions. A minority of individuals obtain formal authorization for medical cannabis access and use. This may reflect federal legality, cost and accessibility of healthcare providers, stigma, scientific evidence supporting its efficacy and physician receptiveness. We did not identify a ‘best’ way of measuring medical cannabis use within a population because this depends on the aims of the study and the research questions being investigated. However, this study indicates caution is needed when drawing conclusions from international comparisons of data on the prevalence of medical cannabis use.

## AUTHOR CONTRIBUTIONS

**Myfanwy Graham**: Conceptualization; formal analysis; project administration; data interpretation; validation; writing—original report; writing—critical revisions; writing—review and editing; final approval. **Rosalie Liccardo Pacula**: Conceptualization; data acquisition; formal analysis; data interpretation; writing—original report; writing—critical revisions; writing—review and editing; final approval. **Seema Choksy Pessar**: Formal analysis; visualization; writing—original report; writing—review and editing; final approval. **Yimin Ge**: Data analysis; data interpretation; writing—editing. **Alexandra F. Kritikos**: Conceptualization; data analysis; writing—original report; writing—review and editing; final approval. **Wayne Hall**: Data interpretation; writing—review and editing; final approval. **David Hammond**: Methodology; project administration; funding acquisition; data acquisition; writing—review and editing; final approval.

## DECLARATION OF INTERESTS

None to declare.

## Supporting information

Table S1. Unweighted counts and weighted proportions of ICPS 2021 survey respondents in total and by country.
Table S2. Multinomial logistic adjusted shares of past‐year cannabis users by measure of use and country.
Table S3. Confidence intervals from multinomial logistic adjusted shares of past‐year cannabis users by measure of use and country.

## Data Availability

The data that support the findings of this study are available from the corresponding author upon reasonable request.
