# Supplementary material for: Understanding medical cannabis use internationally: Why definitions and context matter
Source: Addiction. 2025 Jul 1;120(10):2141–6. doi: 10.1111/add.70117 (PMC12426349; doi:10.1111/add.70117)

**Supplementary Tables**

**Supplemental Table A1: Unweighted Counts and Weighted Proportions of ICPS 2021 Survey Respondents in Total and by Country**

|  | **Entire Sample**  **N (%)** | **Canada**  **N (%)** | **USA**  **N (%)** | **Australia**  **N (%)** |
| --- | --- | --- | --- | --- |
| Total Surveyed | 49,958 | 16,952 | 30,081 | 2,925 |
| Total with no missing gender, age, ethnicity, education | 49,513 | 16,776 | 29,845 | 2,892 |
| Total with information on Past Year Cannabis Use | 32,161 | 10,616 | 20,020 | 1,525 |
| **Total Reporting Past Year Cannabis Use (Our base sample)** | **16,951 (100)** | **5,935 (36.2)** | **10,472 (60.5)** | **544 (3.3)** |
| **Past Year Cannabis Users by Gender** | | | | |
| Female | 10,554 (46.9) | 3,383 (46.6) | 6,906 (47.2) | 265 (44.0) |
| Male | 6,397 (53.1) | 2,552 (53.4) | 3,566 (52.8) | 279 (56.0) |
| **Past Year Cannabis Users by Age Groups** | | | | |
| 16-25 years | 2,446 (18.0) | 717 (16.7) | 1,609 (18.6) | 120 (20.3) |
| 26-35 years | 4,057 (27.8) | 1,521 (28.5) | 2,420 (27.5) | 116 (24.1) |
| 36-45 years | 4,341 (23.0) | 1,448 (23.8) | 2,728 (22.3) | 165 (26.5) |
| 46-55 years | 2,857 (16.7) | 1,002 (16.5) | 1,767 (16.7) | 88 (19.8) |
| 56-65 years | 3,250 (14.6) | 1,247 (14.5) | 1,948 (14.9) | 55 (9.2) |
| **Past Year Cannabis Users by Race** | | | | |
| White | 13,193 (74.6) | 4,405 (72.8) | 8,350 (75.5) | 438 (78.5) |
| Other/Mixed/Unstated | 3,758 (25.4) | 1,530 (27.2) | 2,122 (24.5) | 106 (21.5) |
| **Past Year Cannabis Users by Education** | | | | |
| Refuse to answer | 59 (0.3) | 26 (0.5) | 33 (0.2) | 0 (0.0) |
| Don't know | 56 (0.4) | 16 (0.3) | 38 (0.4) | 2 (0.3) |
| Less than high school | 1,284 (12.1) | 409 (14.8) | 795 (10.0) | 80 (20.6) |
| High school diploma | 3,368 (25.9) | 953 (28.6) | 2,318 (24.4) | 97 (22.1) |
| Some college or technical | 6,861 (37.2) | 2,593 (34.6) | 4,067 (39.0) | 201 (32.8) |
| Bachelor's degree or higher | 5,323 (24.2) | 1,938 (21.3) | 3,221 (25.9) | 164 (24.2) |

**Supplemental Table A2. Multinomial Logistic Adjusted Shares of Past Year Cannabis Users by Measure of Use and Country**


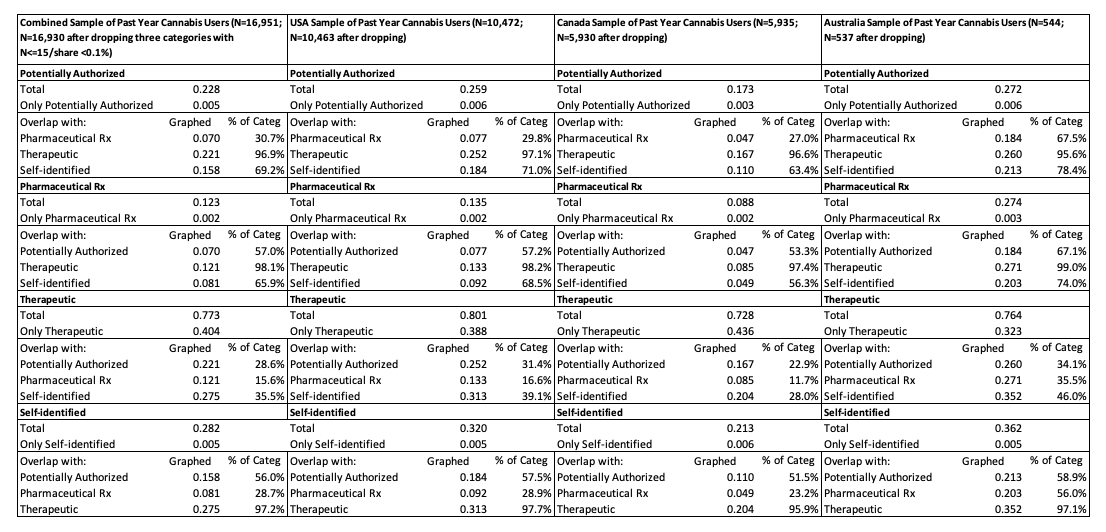


**Supplemental Table A3. Confidence Intervals from Multinomial Logistic Adjusted Shares of Past Year Cannabis Users by Measure of Use and Country**


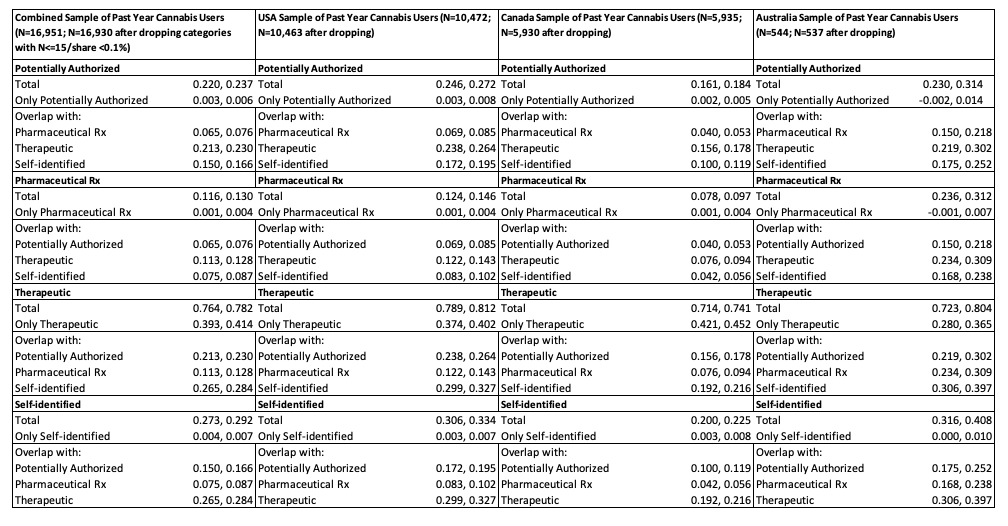

Supplement: Supplementary file 1 — Table S1. Unweighted counts and weighted proportions of ICPS 2021 survey respondents in total and by country. Table S2. Multinomial logistic adjusted shares of past‐year cannabis users by measure of use and country. Table S3. Confidence intervals from multinomial logistic adjusted shares of past‐year cannabis users by measure of use and country. [file ADD-120-2141-s001.docx]
